# Antimicrobial Activity and Molecular Docking Studies of the Biotransformation of Diterpene Acanthoic Acid Using the Fungus *Xylaria* sp.

**DOI:** 10.3390/antibiotics12081331

**Published:** 2023-08-18

**Authors:** Andrey Moacir do Rosario Marinho, Claudia Maria S. C. de Oliveira, João Victor Silva-Silva, Samara C. Anchieta de Jesus, José Edson S. Siqueira, Luana C. de Oliveira, Jéssica Fernandes Auzier, Liviane N. Soares, Maria Lúcia Belém Pinheiro, Sebastião C. Silva, Lívia S. Medeiros, Emmanoel V. Costa, Patrícia S. Barbosa Marinho

**Affiliations:** 1 Post-Graduation in Chemistry, Federal University of Pará, Belém 66075-110, PA, Brazil; samaraibma@gmail.com (S.C.A.d.J.); siqueira.edson@outlook.com (J.E.S.S.); luanaoliveira.qi@gmail.com (L.C.d.O.); pat@ufpa.br (P.S.B.M.); 2 Post-Graduation in Chemistry, Federal University of South and Southeast of Pará, Marabá 68507-590, PA, Brazil; claudiamscosta08@gmail.com (C.M.S.C.d.O.); simotesilva@unifesspa.edu.br (S.C.S.); 3 Laboratory of Medicinal and Computational Chemistry, Institute of Physics of São Carlos, University of São Paulo, São Carlos 13418-900, SP, Brazil; 4 Post-Graduation in Chemistry, Federal University of Amazonas, Manaus 69077-000, AM, Brazil; jessicafauzier27@gmail.com (J.F.A.); bem.liviane@gmail.com (L.N.S.); lbelem1@gmail.com (M.L.B.P.); emmanoelvc@gmail.com (E.V.C.); 5 Post-Graduation in Chemistry, Federal University of São Paulo, Diadema 09920-000, SP, Brazil; livia.soman@unifesp.br

**Keywords:** *Xylaria* sp., biotransformation, diterpenes, acanthoic acid, molecular docking

## Abstract

Biotransformations are reactions mediated by microorganisms, such as fungi. These bioreactions have high chemo- and stereoselectivity on organic substrates and can be applied in the search for new bioactive compounds. In this study, acanthoic acid (AA) was biotransformed using the fungus *Xylaria* sp., giving the novel compound 3β,7β-dihydroxyacanthoic acid (**S1**). Both the AA and the product **S1** were tested against Gram-positive and Gram-negative bacteria. To identify and validate possible biological targets as enzymes or proteins involved in the activity observed in vitro, we used the molecular docking method. Hydroxylation at the C-3 and C-7 positions of the biotransformation product enhanced its activity against *Escherichia coli* as well as its binding affinity and interactions with superoxide dismutase 1 (SOD1; PDB ID 4A7G). Based on our results, the SOD1 enzyme was suggested to be a possible target for the antioxidant activity of product **S1**.

## 1. Introduction

Biotransformation methods are increasingly being used in chemical procedures for the formation of new products. Biotransformation, or biocatalysts, is a chemical process in which an organic compound is subjected to structural modification via whole-cell or isolated enzymes [1,2]. Whole-cell biocatalysts can be used for different types of processes, such as biotransformation, biodegradation, and fermentation. These processes can involve one or more steps to produce the desired chemicals [3,4].

*Xylaria* is one of 40 genera belonging to the Xylariaceae family and is globally distributed. Classification at the species level is difficult because of variations in the coloration, shape, size, and developmental stage [5,6]. Previous studies have demonstrated the enzymatic capacity of fungi for the genus *Xylaria* in biocatalyst processes, such as the biotransformation and biodegradation of the synthetic compounds assisting environmental processes [7] and the biotransformation of saponins [8]. In this context, *Xylaria* is a promising genus in biotransformation methods [5,6].

Bacterial resistance to antibiotics is a serious public health issue and has resulted in the search for new antibiotic compounds, such as diterpenes, to improve the therapeutic arsenal used in the treatment of bacterial infections [9]. Casbane diterpene showed biocidal and biostatic activity against several clinically relevant species (bacteria and yeasts) [10]. Conidiogenone D diterpene, isolated from solid fermentation cultures of the endophytic fungus *Leptosphaeria* sp., showed antibacterial activity against *Bacillus cereus* and *Pseudomonas aeruginosa* [11]. This bioactive potential has been attributed to the structural diversity of diterpenes [12,13,14].

Some diterpenes have been submitted to biotransformation to improve their bioactivity. For example, the fungal biotransformation of *ent*-pimaran-dienoic acid resulted in three products, one of which inhibited the growth of the main microbes responsible for tooth decay [15]. Acanthoic acid (AA) is a pimaradiene diterpene with a wide range of pharmacological activities, such as anti-cancer, anti-inflammatory, anti-diabetes, liver protection, gastrointestinal protection, and cardiovascular protection activity. Structurally modified AA has cytotoxic and anti-inflammatory activity [16,17,18].

Thus, natural compounds modified via biotransformation are promising in the search for new bioactive compounds. In this work, we reported, for the first time, the biotransformation of AA diterpene using the endophytic fungus *Xylaria* sp., leading to the dihydroxylated product 3β,7β-dihydroxyacanoic acid (**S1**).

## 2. Results

### 2.1. Identification of Chemical Constituents

**Figure 1:**
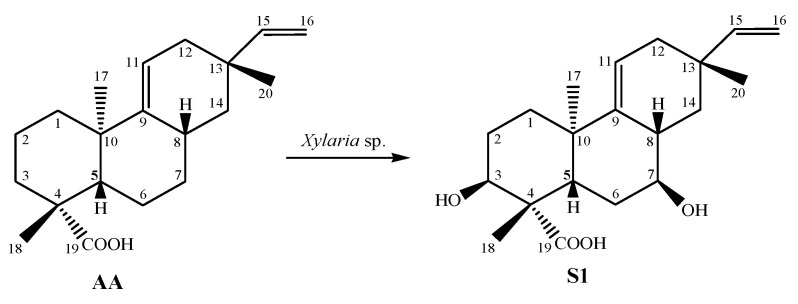
Biotransformation of acanthoic acid (AA) using the fungus *Xylaria* sp. resulting in product **S1**.

The structure of the biotransformation product **S1** (Figure 1) was elucidated using spectroscopic data. Detailed analyses of 1D and 2D NMR (one-dimensional and two-dimensional resonance magnetic nuclear) and MS (mass spectrometry) data were performed. Signal attribution was described according to the results of the spectral analysis and using data from the literature (Table 1).

**Figure 2:**
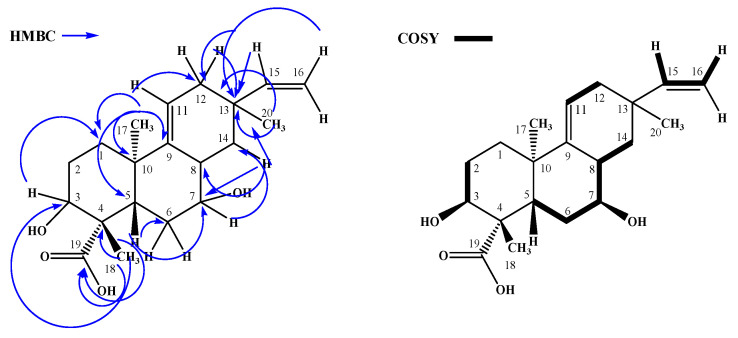
HMBC and COSY correlations to compound **S1**.

Subsequently, the ^1^H NMR values of AA and **S1** were compared, and the absence of the signals for hydrogens H-2, H-3, H-6, and H-7 was observed. An analysis of ^1^H and ^13^C NMR data suggested AA oxidation. Following heteronuclear multiple bond correlation (HMBC) and correlation spectroscopy (COSY), a correlation was observed between all NMR data and compound **S1** (Figure 2).

### 2.2. Antimicrobial Activity

The antimicrobial activity results for both AA and **S1** are presented in Table 2. AA demonstrated activity against *Bacillus subtilis*, with an MIC of 31.25 µg.mL^−1^, while **S1** showed better activity against *Escherichia coli*, with an MIC of 31.25 µg.mL^−1^. Both AA and **S1** exhibited an MIC of 62.5 µg.mL^−1^ against *Salmonella typhimurium*.

### 2.3. Molecular Docking

To understand the difference observed in the antimicrobial activities between substrate AA and product **S1**, without disregarding other possibilities due to the complexity of this system, studies of enzyme regulation were developed through molecular docking using GOLD v. 2020.2.0.

Bactericidal antimicrobials can act by inhibiting DNA synthesis, RNA synthesis, cell-wall synthesis or protein synthesis, and generate reactive oxygen species (ROS) [19,20]. With this in mind, molecular docking simulations were used to show the interaction patterns of AA and **S1** with the penicillin-binding protein 2 protein (PBP2; PDB ID 6G9S) [21], DNA gyrase subunit b (GyrB; PDB ID 4DUH) [22], topoisomerase IV (Topo IV; PDB ID 4HZ0) [23] and superoxide dismutase 1 (SOD1; PDB ID 4A7G) [24] in the hope of finding the mode of action for compounds AA and **S1** based on antimicrobial activity.

To validate the docking configuration, the co-crystallized inhibitors of each protein were redocked into the active sites of FimH, PBP2, GyrB, Topo IV, and SOD1. This validation confirmed the suitability of the docking protocol used for this study. This was demonstrated by the Root Mean Square Deviation (RMSD) values between the experimental pose of the co-crystallized inhibitor and the docked pose of 1.6146 Å in PBP2 (PDB ID 6G9S), 0.5448 Å in GyrB (PDB ID 4DUH), 0.3511 Å in Topo IV (PDB ID 4HZ0), and 0.7531 Å in SOD1 (PDB ID 4A7G), and by the ability of the docking conformation to reproduce all the key interactions achieved by the co-crystallized ligands in the active site.

The analysis of the results from molecular docking simulations revealed that compound AA demonstrated an affinity for the catalytic site region of PBP2 with a docking score value of 50.47. On the other hand, compound **S1** showed a better fit in the catalytic region of SOD1, with a docking score value of 42.45. Moreover, the detailed information and interactions of these ligands with PBP2, SOD1, GyrB, and Topo IV proteins are provided in Table 3.

**Figure 3:**
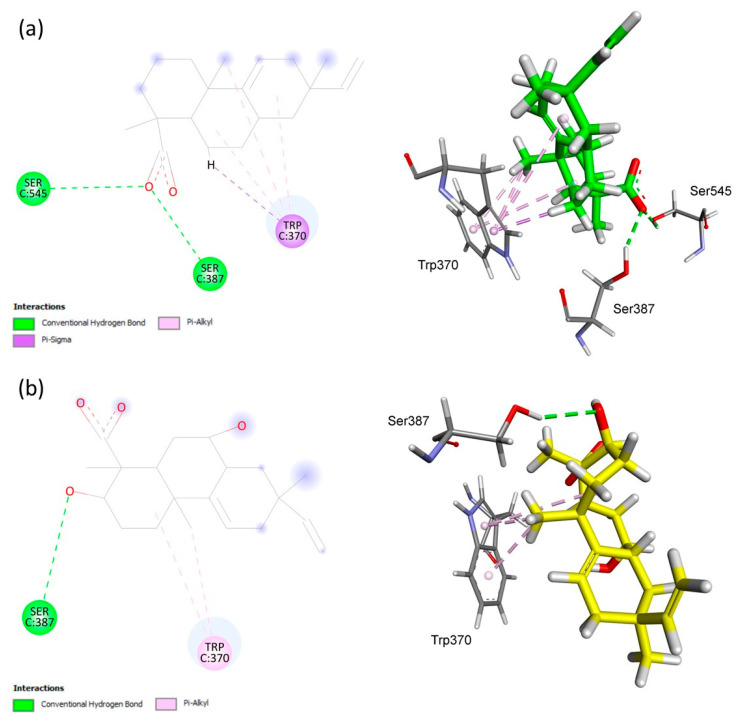
Analysis of docking in the active site of the 6G9S target protein of *Escherichia coli*. (**a**) AA. (**b**) Biotransformation product **S1**. The receptor–ligand interaction is represented on a 2D diagram (**Left**) and a 3D diagram (**Right**). The figure was generated using Biovia Discovery Studio Visualizer software (v. 19.1.0.18287, BIOVIA, San Diego, CA, USA).

**Figure 4:**
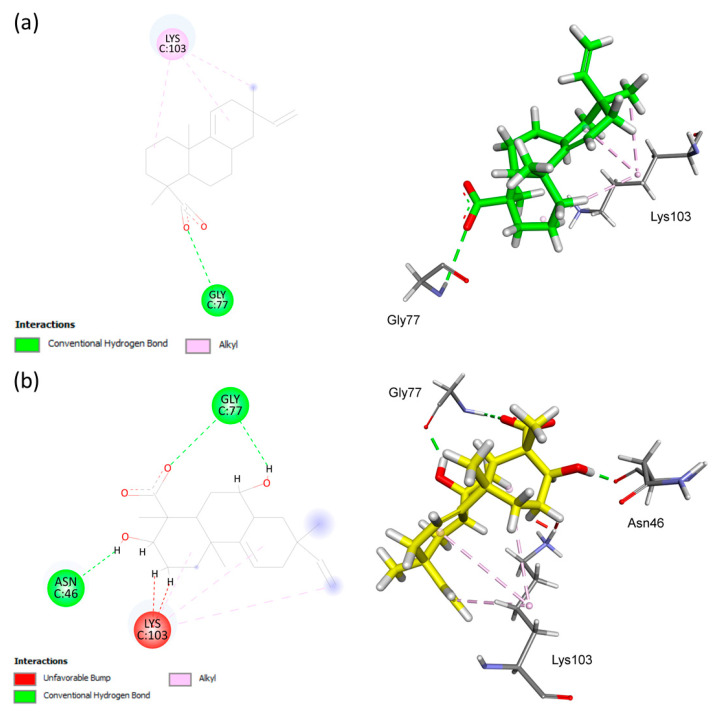
Analysis of the docking in the active site of the 4DUH target protein of *Escherichia coli*. (**a**) Substrate AA. (**b**) Biotransformation product **S1**. The receptor–ligand interaction is represented on a 2D diagram (**Left**) and a 3D diagram (**Right**). This figure was generated using the Biovia Discovery Studio Visualizer software (v. 19.1.0.18287, BIOVIA, San Diego, CA, USA).

In Figure 3, possible chemical interactions that explain the binding of AA and **S1** to the active site of the penicillin-binding protein 2 can be observed. At the Trp370 residue, hydrophobic interactions of the Pi-Alkyl and Pi-Sigma type with aromatic systems occurred. Additionally, there were hydrogen–bonding interactions with the carboxyl functional group of AA. In the case of **S1**, fewer interactions with the active site residues were identified. In this case, the aromatic systems interacted with the methyl group at position C-10, and hydrogen–bonding interactions occurred between Ser387 and the carbonyl group present in the structure of **S1** (Figure 4).

In the binding site of the DNA gyrase subunit B (gyrB) protein, the AA ligand was involved in three alkyl-type hydrophobic interactions with Lys103, while hydrogen–bonding interactions with Gly77 were recognized, interacting with the ligand’s carbonyl group. Furthermore, hydrogen–bonding interactions between Asn46 and Gly77, as well as hydrophobic binding to Lys103 in different regions of **S1**, as shown in Figure 4, were also observed.

**Figure 5:**
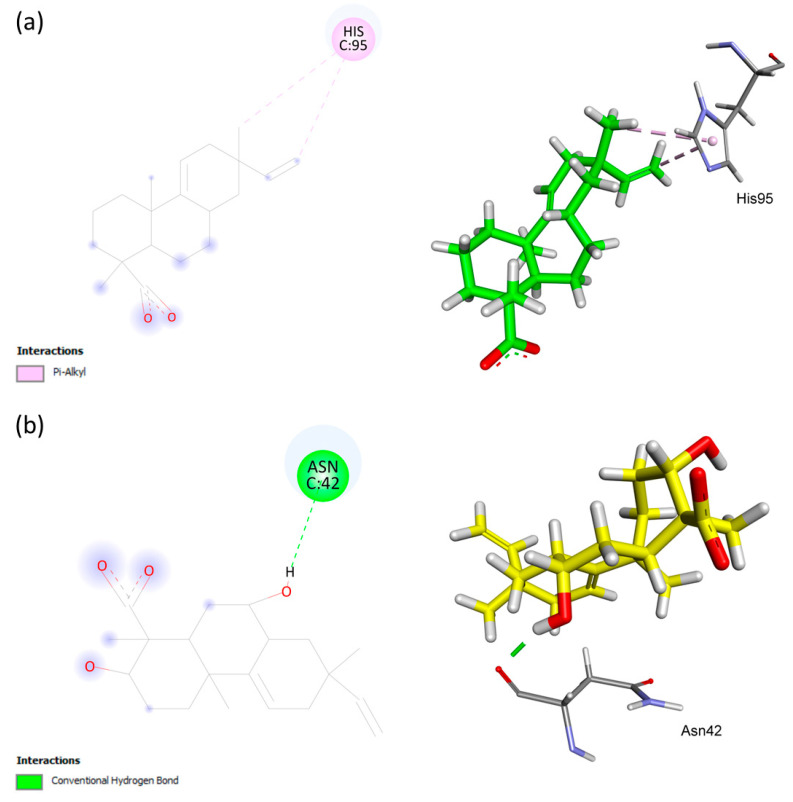
Analysis of the docking in the active site of the 4HZ0 target protein of *Escherichia coli*. (**a**) Substrate AA. (**b**) Biotransformation product **S1**. The receptor–ligand interaction is represented on a 2D diagram (**Left**) and a 3D diagram (**Right**). The figure was generated using Biovia Discovery Studio Visualizer software (v. 19.1.0.18287, BIOVIA, San Diego, CA, USA).

The interactions between AA and **S1** with the residues present in the catalytic site of topoisomerase IV can be observed in Figure 5. Hydrophobic pi–alkyl interactions between His95 and the vinyl group C15=C16 of AA differed from the hydrogen bonding between the residue Asn42 and the carbonyl group of **S1**.

**Figure 6:**
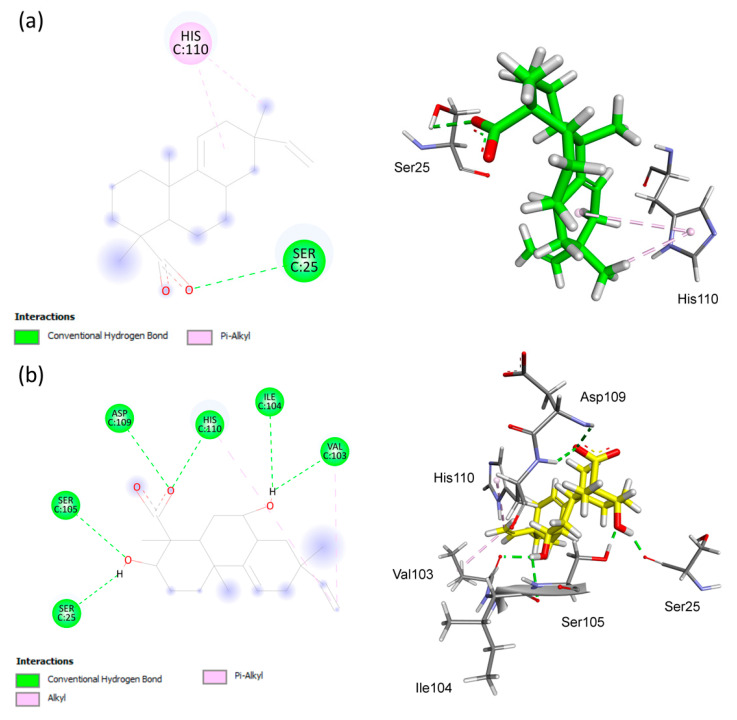
Analysis of the docking in the active site of the 4A7G target protein of *Homo sapiens*. (**a**) Substrate AA. (**b**) Biotransformation product **S1**. The receptor–ligand interaction is represented on a 2D diagram (**Left**) and a 3D diagram (**Right**). The figure was generated using Biovia Discovery Studio Visualizer software (v. 19.1.0.18287, BIOVIA, San Diego, CA, USA).

**Figure 7:**
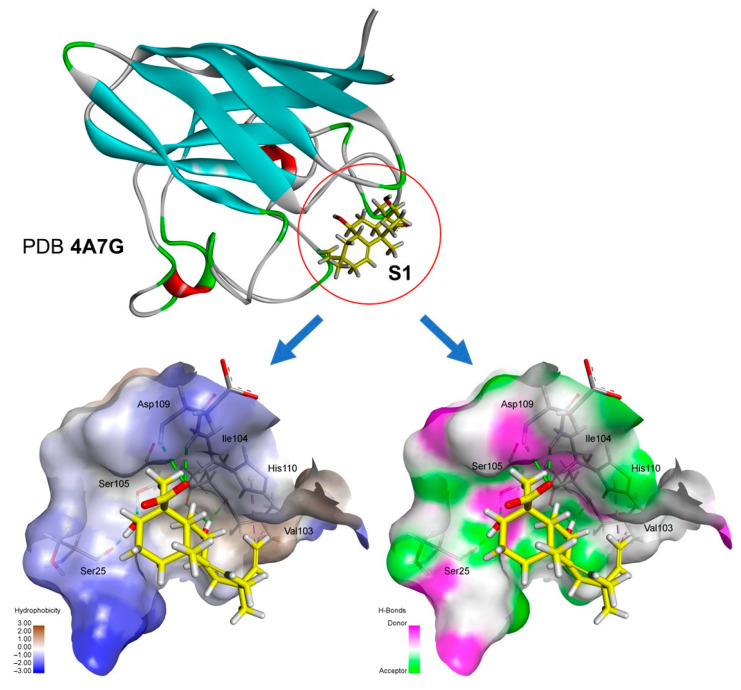
Diagram of hydrophobic surfaces and hydrogen bonds between **S1** and the superoxide dismutase of *Homo sapiens* (PD ID 4A7G). This figure was generated using Biovia Discovery Studio Visualizer software (v. 19.1.0.18287, BIOVIA, San Diego, CA, USA).

Figure 6 illustrates that AA binds tightly through a hydrophobic interaction with the His110 residue, which exhibits the pi–alkyl interaction. Furthermore, AA forms hydrogen–bonding interactions with the Ser25 residue. Both AA and **S1** exhibited a similar binding site with the Ser25 residue, but in the **S1** compound, most of the interactions had shorter distances compared to AA (Table 3). Additionally, **S1** strongly interacted through hydrophobic binding with Val103 (alkyl interaction) and His110 (pi-alkyl and pi–pi stacked interactions) and through hydrogen bonding with Val103, Ile104, Ser105, Asp109, and His110, which was not observed for AA (Figure 7).

To complete the analysis, we reiterated from the hydrophobic surface and hydrogen bond diagrams (Figure 7) that compound **S1** showed effective interactions in the active site of the SOD1 of 4A7G. These results indicate that the structural contrast between AA and **S1**, with hydroxylation at C-3 and C-7 positions of the biotransformation product **S1**, increased the binding.

## 3. Discussion

The high-resolution mass spectrometry–electrospray negative mode, HRMS-ESI (-), spectrum of **S1** showed *m*/*z* 333.2067 [M-H]^-^ and IR spectrum was observed with an OH bond stretching band at 3.423 cm^−1^; these data are compatible to the molecular formula C_20_H_30_O_4_. This translates into two additional hydroxyl groups in the molecule of **S1** compared to AA, which was confirmed by NMR data analysis. A comparison between the ^1^H NMR spectra of AA and **S1** showed chemical shifts at δ_H_ 0.95 (*s*) and δ_H_ 0.97 (*s*), which could be attributed to the methyl groups Me-17 and Me-20, respectively; their signals did not suffer significant changes. For methyl Me-18, we observed an increase in the shift from δ_H_ 1.24 to δ_H_ 1.36 (Δ = +0.12). The signals for vinylic hydrogens of the olefin group were observed as a double doublet (*dd*) at δ_H_ 5.85 (*J* = 17.4 and 10.7 Hz, 1H) to H-15, δ_H_ 4.91 (*cis*, *J* = 10.7 and 1.2 Hz, 1H) and δ_H_ 4.96 (*trans*, *J* = 17.5 and 1.2 Hz, 1H) to H-16a and H-16b. The signals at δ_H_ 1.85 and 2.08 (*J* = 17.4, 4.0 and 2.3 Hz, 1H) were attributed to hydrogens H-12a and H-12b.

Based on the analysis of ^13^C and 2D NMR data, the signals characteristic to methyl groups at δ_C_ 21.6, δ_C_ 23.6, and δ_C_ 22.5 could be attributed to Me-17, Me-18, and Me-20, respectively. The signal at δ 181.0 was typical to the carboxyl group of pimarane diterpenes and was attributed to C-19. For the olefin carbons, we observed signals at δ_C_ 145.7 (C-9), δ_C_ 119.9 (C-11), δ_C_ 149.7 (C-15), and δ_C_ 109.6 (C-16). The chemical shifts in the carbon C-10, C-12, and C-13 to **S1** were similar to those observed for substrate AA. The signal for oximetinic carbon at δ_C_ 70.5 was attributed to C-3, according to the HMBC to Me-18 to *^3^J* HMBC, as well as the HMBC with C-4 (δ_C_ 47.6). In the heteronuclear single quantum coherence (HSQC) spectrum, the signal at δ_H_ 4.12 correlated with δ_C_ 70.5 and was attributed to H-3. The signal at δ_C_ 39.5 was attributed to C-5 based on the HMBC between Me-17 and C-5. The location of the second hydroxylation was defined through the HMBC of H-5 and H-14 with the signal at δ_C_ 72.6 (C-7); this showed the HSQC correlation with signal δ_H_ 3.65, which was to H-7. Moreover, a correlation spin–spin ^1^H–^1^H was observed for H-5, H-6, H-7, H-8, and H-14, confirming that the second hydroxylation was located at the C-7 position. Chemical shifts in the carbon C-1 (δ_C_ 34.8) and C-2 (δ_C_ 27.5) were compared with data from the literature, showing a similarity to pimarane diterpenes with the C-3 position being hydroxylated [25,26,27,28,29]. The NMR, MS and IV spectra for **S1** are available in Supplementary Materials.

The configuration of carbon C-3 and C-7 was determined based on the coupling constant observed. For H-3 (*t*, *J* = 2.8 Hz), equatorial–equatorial coupling with H-2a and H-2b was suggested. The coupling constant for H-7 (*ddd*, *J* = 10.1, 9.0, and 4.9 Hz) showed axial–axial coupling with H-6a and H-8 and axial–equatorial coupling with H-6b. Thus, both OH-3 and OH-7 were β-oriented [30,31,32,33,34]. Therefore, the product **S1**, 3β,7β-dihydroxy-acanthoic acid is novel and reported here for the first time. The main HMBC and COSY correlations for **S1** are shown in Figure 2.

Among the natural secondary metabolites, diterpenoids hold significant importance due to their broad spectrum of antimicrobial activity. In recent years, several in vitro studies have shown that diterpenoid compounds have the capability to inhibit the growth of different strains of antibiotic-resistant bacteria, which have emerged due to the indiscriminate use of antibiotics [35]. Biotransformation enables the generation of a variety of structural analogs that can be evaluated for their antimicrobial activity, thereby expanding the diversity of compounds available for the development of new therapeutic agents against microbial infections [36,37]. In this context, the fungus *Xylaria* sp. was employed to biotransform diterpene acanthoic acid, leading to the introduction of hydroxylation at the C-3 and C-7 positions of the resulting compound **S1**. This modification resulted in a more active molecule against Gram-negative bacteria. Specifically, hydroxylation at these positions improved the antibacterial activity of **S1** against *E. coli* when compared to AA.

Studies have reported that bactericidal antimicrobials have an inhibitory action on DNA synthesis, RNA synthesis, cell wall synthesis, and protein synthesis, as well as generating ROS [19,20]. These antimicrobial activities may involve the inhibition of DNA gyrase (Gyr) and topoisomerase IV (Topo IV), two essential enzymes for bacterial survival, as they perform complementary functions in bacterial DNA processing [38]. Penicillin-binding protein 2 (PBP2) is involved in the inhibition of bacterial cell wall synthesis and belongs to the group of enzymes known as transpeptidases. Penicillin-class antibiotics inhibit transpeptidases, resulting in the disruption of proper bacterial cell wall formation and consequential bacterial death [39]. This mechanism of action demonstrates its overall effectiveness against various bacterial species, including *E. coli* [21]. Additionally, bacteria employ defense mechanisms, such as superoxide dismutase (SOD), to prevent oxidative stress caused by ROS and maintain a cellular redox balance [40].

To obtain a better understanding of the mechanism by which **S1** exhibited superior inhibition against *E. coli*, in silico enzymatic evaluation assays were developed using molecular docking studies. This molecular docking technique was highly useful in determining the binding affinity of drug molecules to the biological target [41]. In this case, the docking simulations evaluated the binding patterns of diterpenoids at the active sites of proteins 6G9S (PBP2), 4DUH (GyrB), 4HZ0 (Topo IV), and 4A7G (SOD1).

In this study, a difference in affinity between AA and **S1** for the active site region of the analyzed proteins was observed. An analysis of the molecular docking simulations revealed that AA showed an affinity for the catalytic region of PBP2 (PDB ID 6G9S), while **S1** exhibited a better fit in the catalytic region of SOD1 (PDB ID 4A7G). It can be observed that AA interacts with residues Trp370, Ser387, and Ser545, which are part of the active site structure of PBP2 from *E. coli* (Ser330, Thr331, Val332, Lys333, Arg368, Asp369, Trp370, Lys371, Ser387, Ala388, Asp389, Ile453, Gly454, Gln455, Gly456, Lys544, Ser545, Gly546, and Thr547) [21]. This result suggests that the inhibition pathway of AA likely has an affinity for the same active site region as penicillin-class antibiotics [39], indicating its role in disrupting the proper formation of the bacterial cell wall. The docked pose clearly showed that both AA and **S1** bound within the active site of the SOD1; however, the binding mode was improved in **S1** because of significant hydrogen bonding within the catalytic active site with Ser25, Val103, Ile104, Ser105, Asp109, and His110. This result suggests that **S1** inhibits *E. coli* via the superoxide dismutase pathway. Hydrogen bonds play a central role in protein–ligand binding affinity, including enzymatic catalysis, to stabilize a ligand in a binding pocket [42,43]. Published results regarding the antimicrobial activity of pimarane diterpenes against different bacteria indicate that the presence of a hydrogen-bond donor group (HBD) is important for the antimicrobial activity of diterpenes. Moreover, the distance between HDB groups can interfere with antimicrobial activity [44]. Based on the observations above, we could assume that **S1** showed improved activity against *E. coli* because of additional HDB groups at C-3 and C-7, favoring the chemical interactions between **S1** and the protein target by the formation of a stable enzyme–substrate complex.

Therefore, molecular docking simulations provided a clearer understanding of the mechanisms of action for diterpenes AA and **S1** on target proteins, revealing valuable insights into their interactions at the active site. These results contribute to a better comprehension of the results obtained in the in vitro tests. However, further experimental studies are needed to confirm the proposed association between SOD1 and the observed antioxidant activity of **S1**.

## 4. Materials and Methods

### 4.1. Microorganisms

The fungus *Xylaria* sp. (code EJCP07) was obtained from the collection of the Laboratory of Bioassays and Chemistry of the Microorganisms (LaBQuiM), Federal University of Pará, Brazil. It was reactivated for 7 days in a Petri dish containing BDA medium to be used in the biotransformation reactions in this work.

### 4.2. Acanthoic Acid

The diterpene used as a substrate in this work was an authentic sample of acanthoic acid (AA) isolated from *Annona amazonica* (Annonaceae) in a previous study [45].

### 4.3. Biotransformation Reactions Procedure

Biotransformation was performed in five 500 mL Erlenmeyer flasks, each containing 200 mL of the Czapek medium. The flasks were autoclaved for 15 min at 121 °C and 1 atm pressure. Subsequently, three small disks of 2 mm^2^, containing *Xylaria* sp. (EJCP07) mycelium, were added to two Medium + Fungus + Substrate (MFS) flasks and used for biotransformation with one Medium + Substrate (MS) flask (control). The Medium (M), Medium + Fungus (MF) flasks were also used as control. The flaks were placed on an orbital shaker (Quimis Q315IA) at 32 °C and 150 rpm. After 3 days, 30 mg (per flask) of the AA was solubilized in DMSO and added to flasks MFS and MS. The flaks remained on the shaker for another 5 days, and after this, the mixtures were filtered to obtain the mycelium and the aqueous phase. The mycelium was discarded, and the aqueous phase was used for liquid–liquid extraction with ethyl acetate (EA) (3 × 100 mL). After this, the EA phase was dried with anhydrous sodium sulfate (Na_2_SO_4_), filtered, and concentrated in a rotary evaporator to obtain the EA phase of the biotransformation extract (PEABE).

### 4.4. Fractionation of PEABE Extract and Biotransformation Product Purification

We obtained 40.5 mg of the PEABE extract, which was submitted to an exclusion column chromatograph Sephadex LH-20 eluted with methanol, resulting in 11 fractions (Fr_1_–Fr_11_). The fractions were analyzed via TLC and ^1^H NMR, and the biotransformation product was detected in fraction Fr_5_ (16.7 mg). This fraction was then fractionated using a silica gel column chromatography, increasing the polarity gradient elution of Hex/EtAcO 40% (100 mL), Hex/EtAcO 60% (60 mL), EtAcO 100% (50 mL), EtAcO/MeOH (50%) and MeOH 100% (50 mL), which resulted in 19 fractions (Frs_1_–Frs_19_). After TLC, similar fractions were pooled, and the biotransformation product **S1** was obtained (1.7 mg).

### 4.5. Characterisation of the Compounds

The 1D and 2D NMR spectra were obtained using a Bruker Ascend 400 (400 MHz) nuclear magnetic resonance (NMR) spectrometer (Bruker, Fällanden, Switzerland) with chloroform-d as the solvent. The HRMS values were obtained on a MicroTOF-QII (Bruker Daltonics, EUA) equipped with an electrospray source (ESI) operating in the negative ion mode.

### 4.6. Antimicrobial Assay

The antimicrobial susceptibility test was carried out using the microbroth dilution assay [46]. Tests were performed on 96-well plates with 100 μL of Mueller–Hinton broth (MHB) (HiMedia, Mumbai, India), 100 μL of the test compound, and 5 μL of test bacteria at 1.0 × 10^4^ CUF/mL, followed by incubation at 37 °C (24 h). The compounds were dissolved (initially 1 mg) in 100 μL of dimethyl sulfoxide (DMSO) and 900 μL of the brain heart infusion (BHI) broth, resulting in a 1 mg/mL stock solution. The stock solution was diluted from 500 to 7.81 µg/mL for testing. *E. coli* (ATCC 25922), *S. typhimurium* (ATCC 14028), and *B. subtilis* (ATCC 6633) were provided by Evandro Chagas Institute, Belém, Pará State, Brazil. Bioactivity was registered as the absence of red coloration in the wells after the addition of 10 μL of 2,3,5-triphenyltetrazolium chloride. The microorganisms were then sub-cultured on MHB plates. The activities of the test compounds were classified as bacteriostatic or bactericidal according to the behavior of the microorganisms in these sub-cultures. Amoxicillin and terramycin were used as positive controls, and the MHB culture medium was used as a negative control.

### 4.7. Molecular Docking

The molecules (AA and the biotransformation product **S1**) were drawn using ChemDraw Professional 16.0 and were optimised via Avogadro (version 1.2.0) with a UFF (Universal Force Field) up to dE = 1 × 10^−12^ kJ/mol and exported as mol2 [47]. Molecular docking simulations were performed using the GOLD v. 2022.1 program, which is available free of charge from CSDS (bdec.dotlib.com.br/inicio_csds/application/Hermes (accessed on 25 February 2022)). Four different scoring functions (ASP, ChemScore, GoldScore and ChemPLP) were applied in enzyme–substrate interaction calculations, as described by Silva-Silva et al. [48]. Protein structures (PDB ID 6G9S, 2.00 Å; PDB ID 4DUH, 1.50 Å; PDB ID 4HZ0, 2.20 Å; PDB ID 4A7G, 1.24 Å) were treated as rigid, and the compounds were treated as fully flexible. Only chain A of the receptor was used, and no crystallographic water molecules were considered [49,50]. The binding site was defined as all receptor atoms up to 10 Å of the reference crystallographic inhibitor. At least 10 poses were generated for the ligand, using the default parameters of the genetic algorithm. The validation process of the applied calculation was carried out through redocking studies, evaluating the RMSD value of the co-crystallised ligand pose, which presented the best score value and its alignment between the generated poses. The results were visually analysed with the help of Discovery Studio Visualizer v. 19.1.0.18287 (BIOVIA, San Diego, CA, USA). The 2D and 3D enzyme–substrate interaction diagrams were produced in Discovery Studio Visualizer v. 19.1.0.18287.

## 5. Conclusions

The biotransformation of AA using the fungus *Xylaria* sp. led to the formation of a new compound, 3β,7β-dihydroxy-acanthoic acid (**S1**), with improved activity against *E. coli* compared to AA. In docking studies, **S1** showed promising characteristics and can, therefore, be used to design new antibacterial agents that are effective against protein superoxide dismutase 1.

## Figures and Tables

**Table 1:** ^1^H and ^13^C NMR (400 MHz, CDCl_3_) data for compounds AA and **S1**.

	AA		**S1**	
n	^1^H δ (Mult, *J* in Hz)	^13^C	^1^H δ (Mult, *J* in Hz)	^13^C
1	ax 1.28 (*m*) eq 1.79 (*m*)	41.9	1.55 1.70	34.8
2	ax 2.19 (*m*) eq 1.92 (*m*)	18.9	1.68 (*m*) 2.22 (*m*)	27.5
3	ax 1.05 (*m*) eq 2.15 (*m*)	38.1	4.12 (*t*, 2.8)	70.5
4	-	44.2	-	47.6
5	1.66 (*dd*, 12.9 and 6.0)	48.0	2.30 (*dd*, 12.8 and 5.0)	39.5
6	ax 1.48 (*m*) eq 1.90 (*m*)	20.3	1.70 (*m*) 2.75 (*m*)	30.8
7	ax 1.21 (*m*) eq 1.73 (*m*)	27.8	3.65 (*ddd*, 10.1, 9.0 and 4.9)	72.6
8	2.32 (*m*)	28.7	2.40 (*m*)	37.2
9	-	149.9	-	145.7
10	-	38.4	-	38.2
11	5.39 (*dt*, 5.0 and 2.1)	116.6	5.53 (*dt*, 5.3 and 2.2)	119.9
12	ax 1.75 (*m*) eq 2.03 (*ddd*, 17.4; 4.0 and 2.3)	37.5	1.85 (*m*) 2.08 (*ddd*, 17.4; 4.0 and 2.3)	37.3
13	-	34.8	-	34.4
14	ax 1.02 (*m*) eq 1.45 (*m*)	41.8	1.15 (*m*) 1.26 (*m*)	38.7
15	5.81 (*dd*, 17.5 and 10.7)	150.2	5.86 (*dd*, 17.4 and 10.7)	149.7
16	4.86 *cis* (*dd*, 10.7 and 1.4) 4.93 (*dd*, 17.5 and 1.4)	109.1	4.92 (*dd*, 10.7 and 1.2) 4.95 (*dd* 17.5 and 1.2)	109.6
17	0.96 (*s*)	22.2	0.95 (*s*)	21.6
18	1.24 (*s*)	28.5	1.37 (*s*)	23.6
19	-	184.3	-	181.0
20	0.99 (*s*)	22.4	0.98 (*s*)	22.5

**Table 2:** Antimicrobial activity of substrate and biotransformation product.

Compound	MIC (µg.mL^−1^)		
	Gram (+) Bacteria	Gram (−) Bacteria	
	*Bacillus subtilis* (ATCC 6633)	*Escherichia coli* (ATCC 25922)	*Salmonella typhimurium* (ATCC 14028)
AA	31.25	250	62.5
**S1**	500	31.25	62.5
Amoxicillin	7.81	7.81	7.81
Tetracycline	7.81	7.81	7.81

MIC: minimum inhibitory concentration; AA: acanthoic acid; **S1**: biotransformation product.

**Table 3:** Docking score and interactions of AA and biotransformation product **S1** after docking at the penicillin-binding protein 2 (PDB ID 6G9S), DNA gyrase (PDB ID 6G9S), topoisomerase IV (PDB ID 4HZ0) and superoxide dismutase (PDB ID 4A7G).

Compounds	Docking Score	Bond Category	Residues in Contact	Interaction Types	Distance (Å)
Docking inside the penicillin-binding protein 2 (PDB: 6G9S)					
AA	Goldscore fitness: 50.47	Hydrophobic	TRP370	PA	4.02, 4.05, 5.11, 5.48
		Hydrophobic	TRP370	PS	2.55
		Hydrogen	SER387	H	2.43
		Hydrogen	SER545	H	2.54
**S1**	Goldscore fitness: 44.29	Hydrophobic	TRP370	PA	3.20, 3,70, 5.15
		Hydrogen	SER387	H	2.87
ET5	Goldscore fitness: 49.58	Hydrogen	SER330	H	2.23, 2.88
		Hydrogen	LYS333	H	3.02
		Hydrophobic	TRP370	PA	4.98
		Hydrophobic	TRP370	PPS	5.01
		Unfavorable	TYR393	UDD	2.38
		Hydrogen	ILE453	H	2.35
		Hydrogen	GLN455	H	2.50
		Hydrogen	LYS544	H	5.18
		Hydrogen	THR547	H	1.86
Docking inside the DNA gyrase subunit B (PDB: 4DUH)					
AA	ChemPLP Score: 50.62	Hydrophobic	GLY77	A	2.88
		Hydrogen	LYS103	H	3.81, 4.08, 5.43
**S1**	ChemPLP Score: 40.70	Hydrogen	ASN46	H	1.71
		Hydrogen	GLY77	H	1.76, 2.70
		Hydrophobic	LYS103	UB	1.26, 1.33
		Hydrophobic	LYS103	A	4.36, 5.13, 5.15
RLI	ChemPLP Score: 78.99	Hydrophobic	VAL43	A	4.70
		Hydrogen	ASN46	H	3.03
		Hydrophobic	VAL71	A	4.17
		Hydrogen	ASP73	H	1.70
		Hydrogen	ARG76	H	2.56
		Hydrophobic	ILE78	PA	4.65, 4.90, 4.99
		Hydrophobic	PRO79	PA	4.14
		Hydrophobic	ILE94	PA	4.84
		Hydrophobic	LYS103	PA	4.64
		Hydrogen	ARG136	H	1.80, 2.10
		Hydrophobic	VAL167	A	4.21
Docking inside the topoisomerase IV (PDB: 4HZ0)					
AA	ChemPLP Score: 41.28	Hydrophobic	HIS95	PA	3.82, 4.18
**S1**	ChemPLP Score: 43.29	Hydrogen	ASN42	H	1.98
1AV	ChemPLP Score: 57.00	Hydrophobic	ASN42	APS	4.67
		Hydrogen	ASP69	H	1.85
		Hydrophobic	ARG72	PA	5.48
		Hydrophobic	MET74	PS	2.76
		Hydrophobic	MET74	PA	4.15, 4.74
		Hydrophobic	PRO75	PA	4.06
		Hydrophobic	ILE90	PA	4.51
Docking inside the superoxide dismutase (PDB: 4A7G)					
AA	ChemPLP Score: 31.34	Hydrogen	SER25	H	2.61
		Hydrophobic	HIS110	PA	4.26, 5.14
**S1**	ChemPLP Score: 42.45	Hydrogen	SER25	H	1.68
		Hydrophobic	VAL103	A	3.76
		Hydrogen	VAL103	H	2.02
		Hydrogen	ILE104	H	2.99
		Hydrogen	SER105	H	1.89
		Hydrogen	ASP109	H	2.39
		Hydrophobic	HIS110	PA	4.58
		Hydrogen	HIS110	H	2.75
12I	ChemPLP Score: 41.74	Hydrogen	ASP109	H	2.55
		Hydrogen	HIS110	H	2.13
		Hydrophobic	HIS110	PPS	3.68, 4.31

AA: acanthoic acid; **S1**: biotransformation product; KGM: (2S,3S,4S,5S,6R)-2-heptoxy-6-(hydroxymethyl)oxane-3,4,5-triol; ET5: (3R,6S)-6-(aminomethyl)-4-(1,3-oxazol-5-yl)-3-(sulfooxyamino)-3,6-dihydro-2H-pyridine-1-carboxylic acid; RLI: 4-[[4-[4-methyl-2-(propanoylamino)-1,3-thiazol-5-yl]-1,3-thiazol-2-yl]amino]benzoic acid; 1AV: 7-imidazol-1-yl-2-pyridin-3-yl-[1,3]thiazolo[5,4-d]pyrimidin-5-amine; 12I: 4-(4-methylpiperazin-1-yl)quinazoline; A: Alkyl; APS: Amide-Pi Stacked; H: conventional hydrogen bond; PA: Pi-Alkyl; PPS: Pi-Pi Stacked; PS: Pi-Sigma; UDD: Unfavourable donor-donor; UB: Unfavourable Bump.

## Data Availability

The data presented in this study are available in the Supplementary Material.

## Supplementary Materials

The following supporting information can be downloaded at: https://www.mdpi.com/article/10.3390/antibiotics12081331/s1, Figures S1–S7: NMR, IR, and MS spectra of the active compound are available online.

## Sample Availability

Samples of the compounds are available from the authors.
