# Supplementary material for: Antimicrobial Activity and Molecular Docking Studies of the Biotransformation of Diterpene Acanthoic Acid Using the Fungus Xylaria sp
Source: Antibiotics (Basel). 2023 Aug 18;12(8):1331. doi: 10.3390/antibiotics12081331 (PMC10451833; doi:10.3390/antibiotics12081331)
Supplement: Supplementary file 1 [file antibiotics-12-01331-s001.zip › antibiotics-2510169-supplementary.pdf]

Supplementary Information

Antimicrobial Activity and Molecular Docking Studies of the Biotransformation of Diterpene Acanthoic Acid Using the Fungus Xylaria sp.

Andrey Moacir do Rosario Marinho [1,*], Claudia Maria S. C. de Oliveira [2], João Victor Silva-Silva [3,*], Samara C. Anchieta de Jesus [1], José Edson S. Siqueira [1], Luana C. de Oliveira [1], Jéssica Fernandes Auzier [4], Liviane N. Soares [4], Maria Lúcia Belém Pinheiro [4], Sebastião C. Silva [2], Lívia S. Medeiros [5], Emmanoel V. Costa [4] and Patrícia S. Barbosa Marinho [1]

[1] Post-Graduation in Chemistry, Federal University of Pará, Belém 66075-110, PA, Brazil; samaraibma@gmail.com (S.C.A.d.J.); siqueira.edson@outlook.com (J.E.S.S.); luanaoliveira.qi@gmail.com (L.C.d.O.); pat@ufpa.br (P.S.B.M.)

[2] Post-Graduation in Chemistry, Federal University of South and Southeast of Pará, Marabá 68507-590, PA, Brazil; claudiamscosta08@gmail.com (C.M.S.C.d.O.); simotesilva@unifesspa.edu.br (S.C.S.)

[3] Laboratory of Medicinal and Computational Chemistry, Institute of Physics of São Carlos, University of São Paulo, São Carlos 13418-900, SP, Brazil

[4] Post-Graduation in Chemistry, Federal University of Amazonas, Manaus 69077-000, AM, Brazil; jessicafauzier27@gmail.com (J.F.A.); bem.liviane@gmail.com (L.N.S.); lbelem1@gmail.com (M.L.B.P.); emmanoelvc@gmail.com (E.V.C.)

[5] Post-Graduation in Chemistry, Federal University of São Paulo, Diadema 09920-000, SP, Brazil; livia.soman@unifesp.br

* Correspondence: andrey@ufpa.br (A.M.d.R.M.); jvssilva89@gmail.com (J.V.S.-S.)

Figure S1. ^{1}H NMR spectrum (400 MHz, $CDCl_3$) of compound **S1**. A) full spectrum; B) Expansion 2.00 to 6.00 ppm; C) Expansion 0.00 to 3.00 ppm

Figure S2. ^{13}C NMR spectrum (100 MHz, $CDCl_3$) of compound **S1**. A) full spectrum; B) Expansion 10.0 to 75.0 ppm

Figure S3. HSQC spectrum of **S1** ($CDCl_3$, 400 MHz). A) full spectrum; B) Expansion

Figure S4. HMBC spectrum of **S1** ($CDCl_3$, 400 MHz). A) Full spectrum; B) Expansion

Figure S5. COSY spectrum of **S1** ($CDCl_3$, 400 MHz). A) Full spectrum; B) Expansion

Figure S6. Mass spectrum HR(ESI) negative ion mode of compound **S1**.

Figure S7. IR spectrum of compound **S1**.

This is an open-access article distributed under the terms of the Creative Commons Attribution Licence.

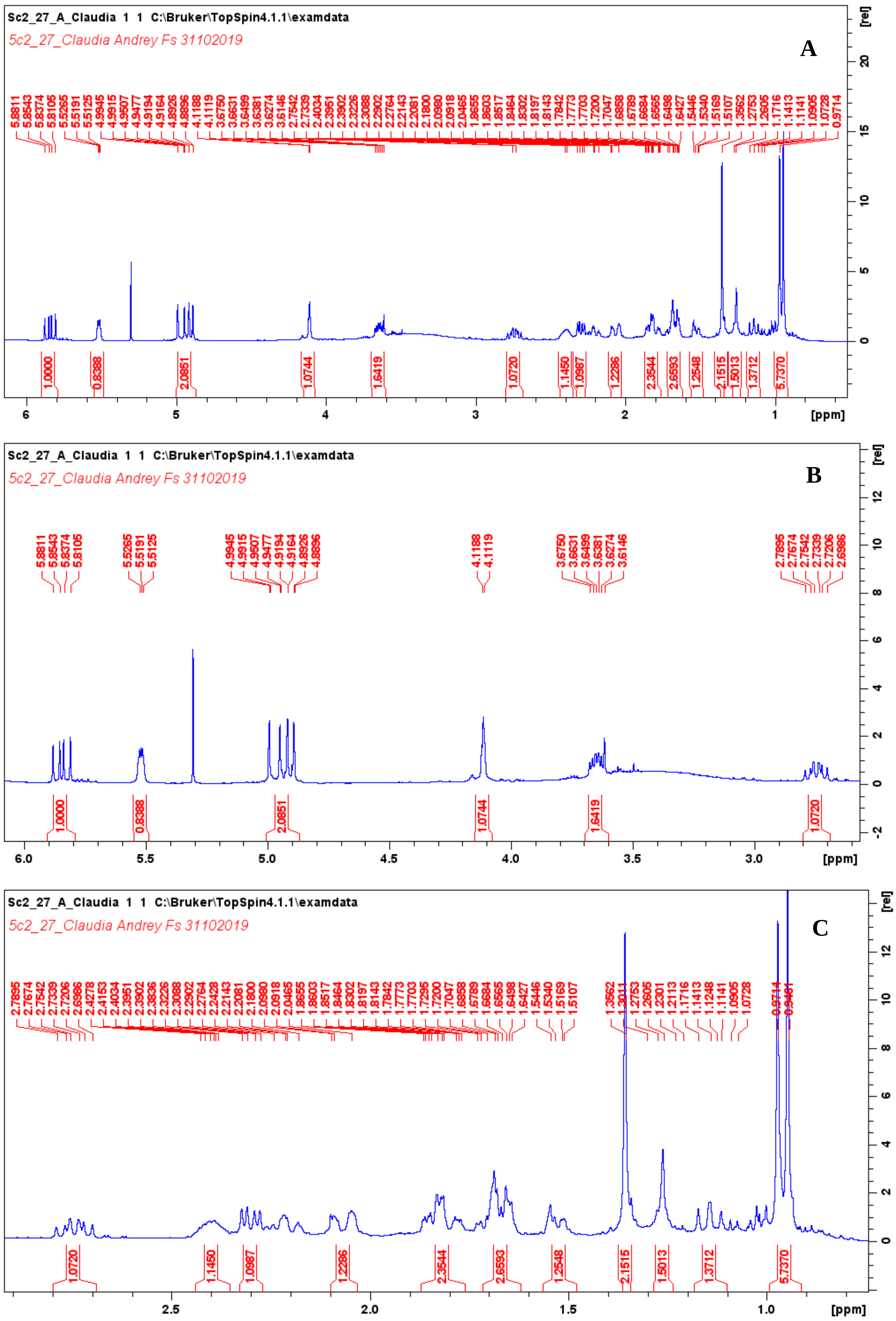

Figure S1. ^{1}H NMR spectrum (400 MHz, $CDCl_3$) of compound **S1**. A) full spectrum; B) Expansion 2.00 to 6.00 ppm; C) Expansion 0.00 to 3.00 ppm

This is an open-access article distributed under the terms of the Creative Commons Attribution Licence.

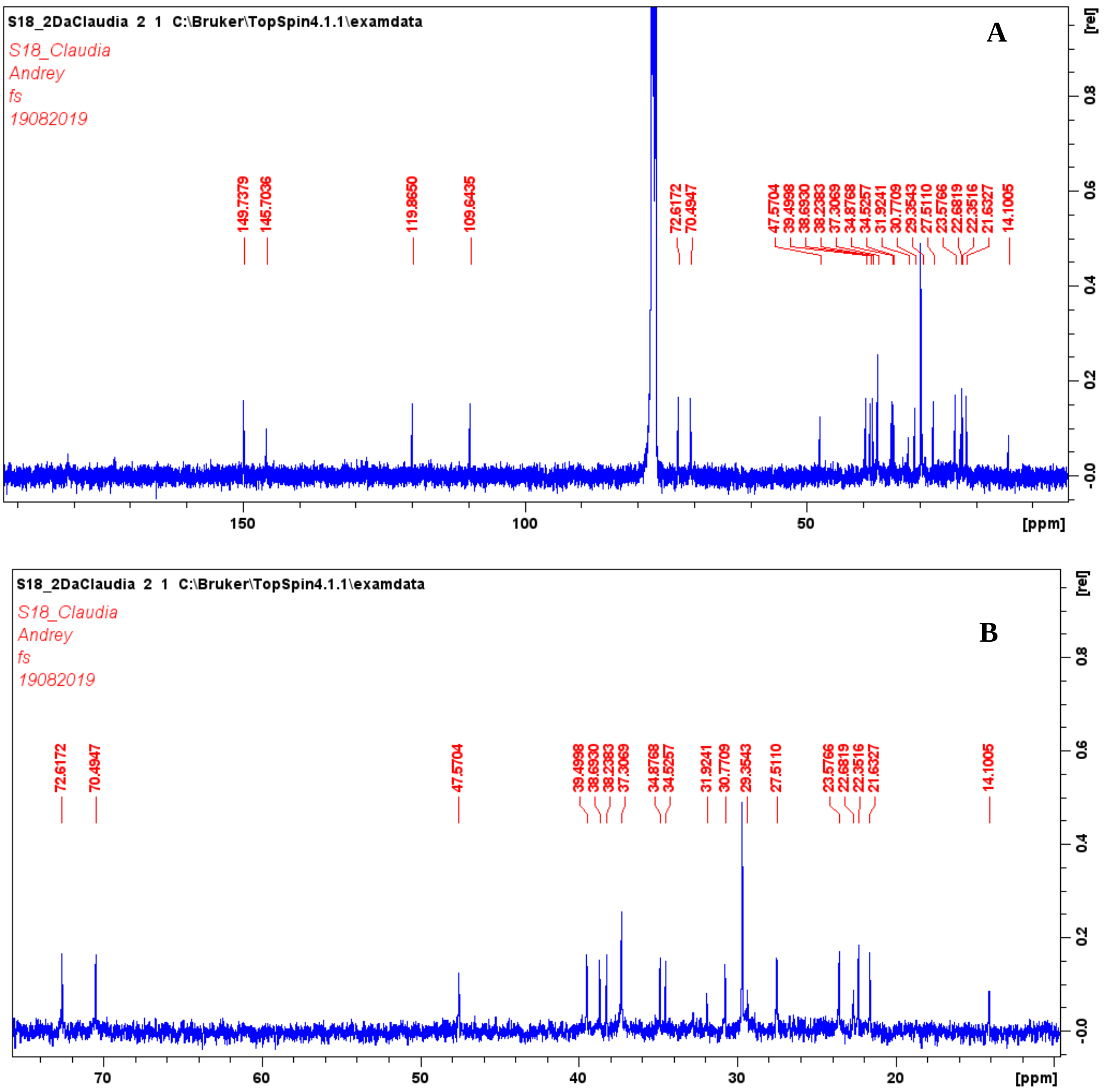

Figure S2. ^{13}C NMR spectrum (100 MHz, $CDCl_3$) of compound **S1**. A) full spectrum; B) Expansion 10.0 to 75.0 ppm

This is an open-access article distributed under the terms of the Creative Commons Attribution Licence.

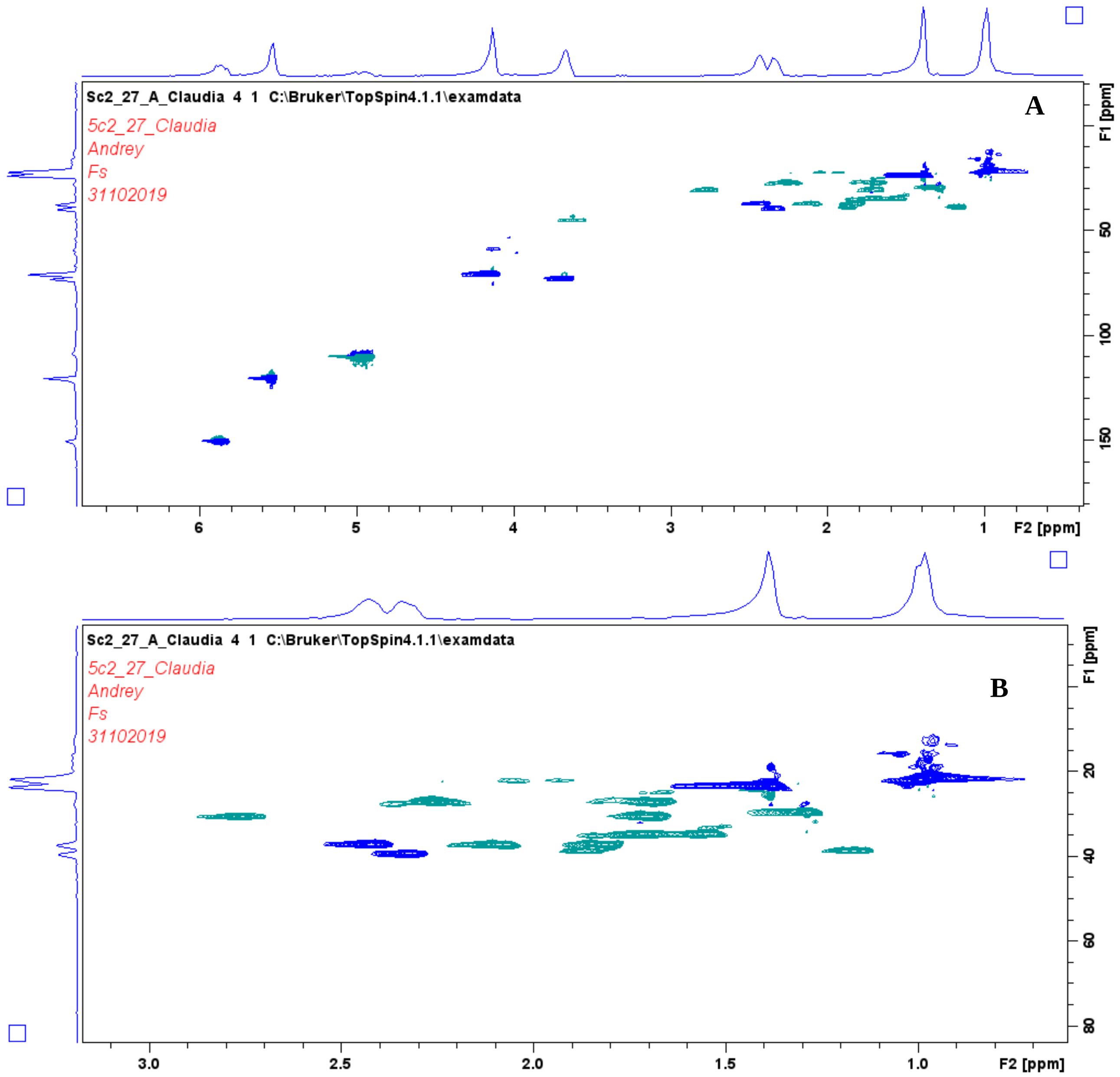

Figure S3. HSQC spectrum of **S1** ($CDCl_3$, 400 MHz). A) full spectrum; B) Expansion

This is an open-access article distributed under the terms of the Creative Commons Attribution Licence.

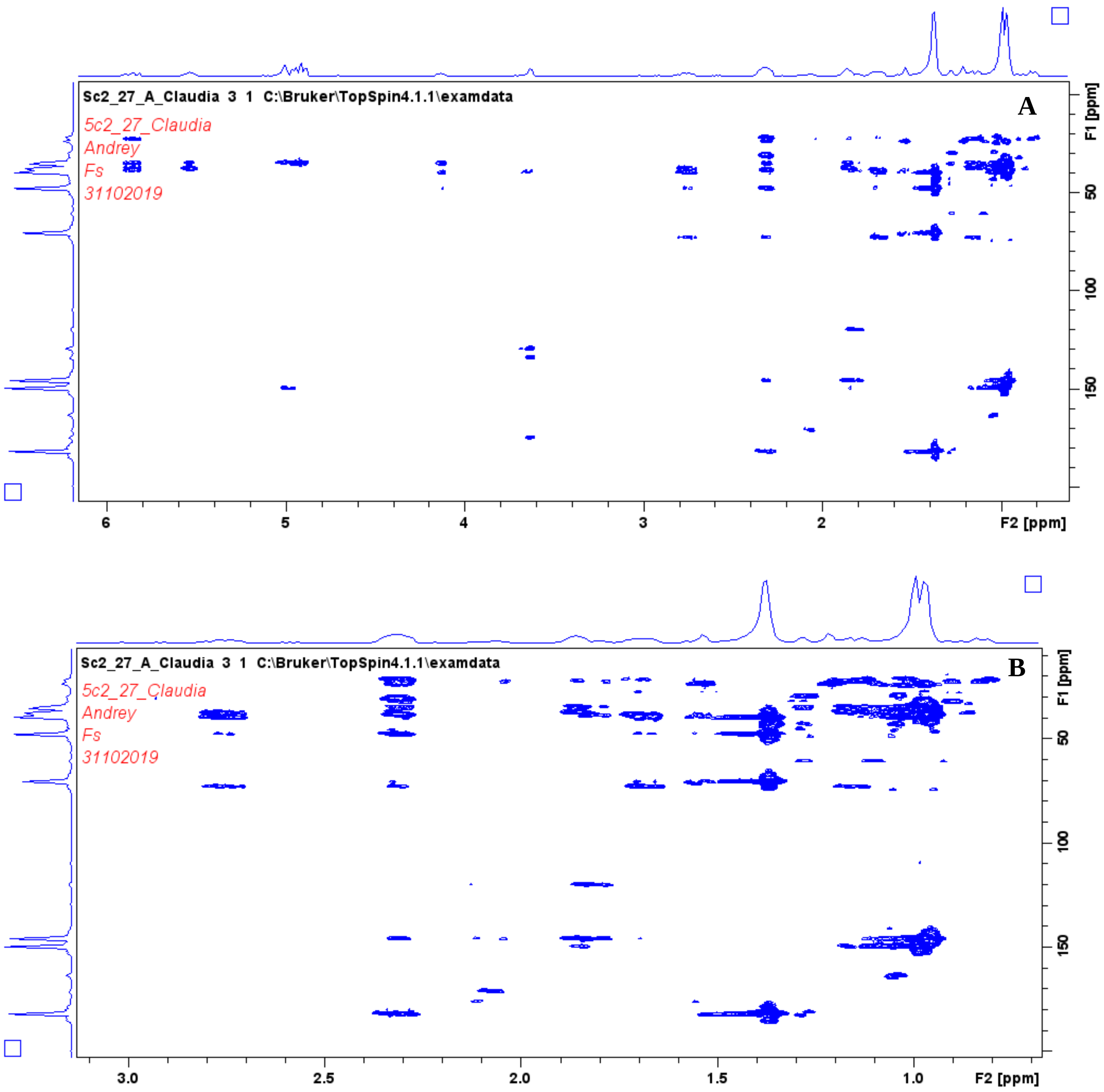

Figure S4. HMBC spectrum of **S1** ($CDCl_3$, 400 MHz). A) Full spectrum; B) Expansion

This is an open-access article distributed under the terms of the Creative Commons Attribution Licence.

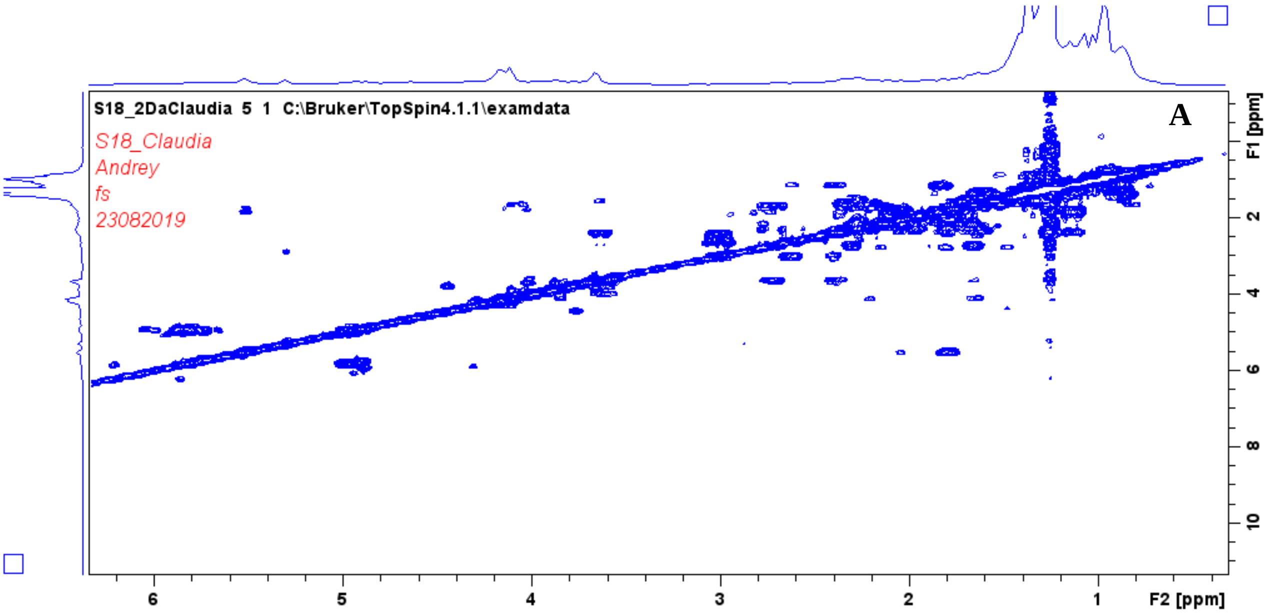

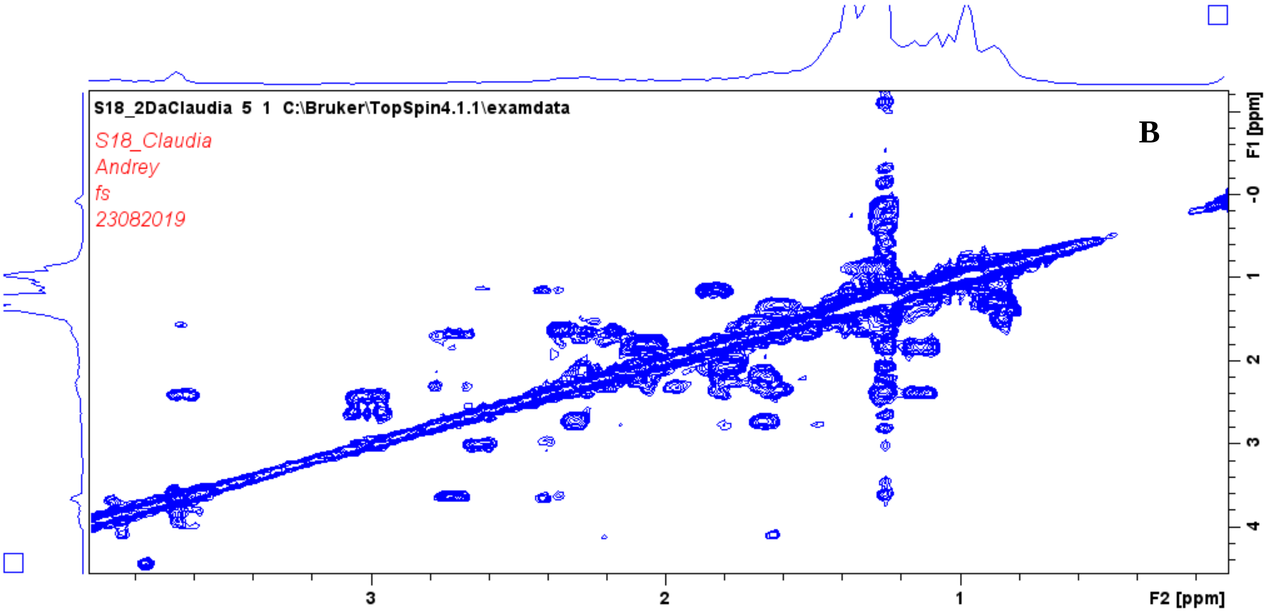

Figure S5. COSY spectrum of **S1** ($CDCl_3$, 400 MHz). A) Full spectrum; B) Expansion

This is an open-access article distributed under the terms of the Creative Commons Attribution Licence.

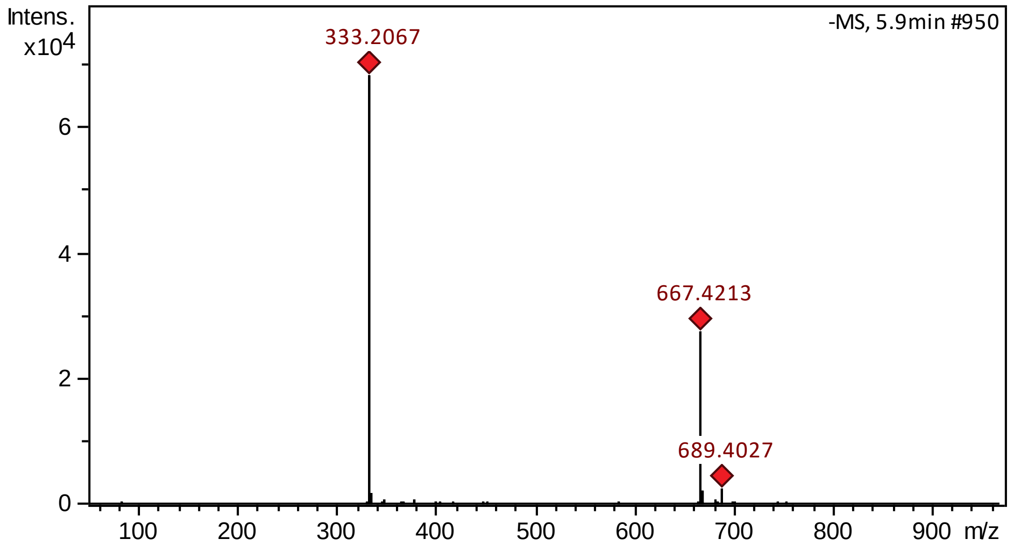

Figure S6. Mass spectrum HR(ESI) negative ion mode of compound **S1**.

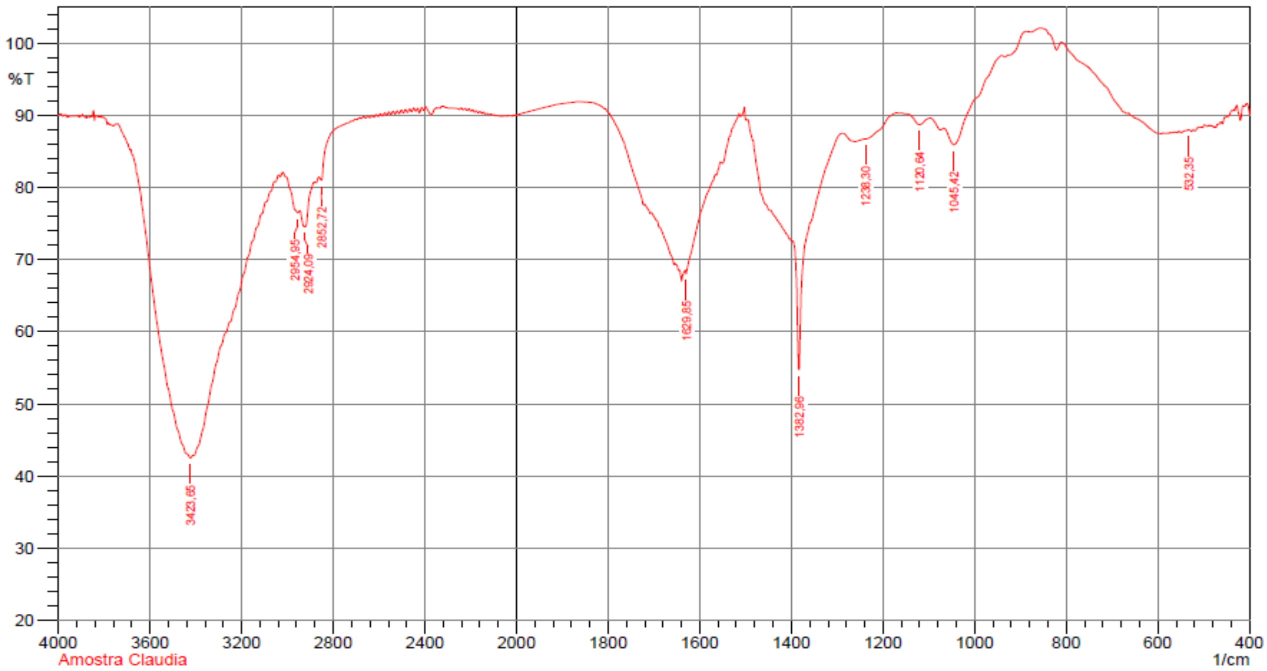

Figure S7. IR spectrum of compound **S1**.

This is an open-access article distributed under the terms of the Creative Commons Attribution Licence.
